# Injecting Electrons into CeO_2_ via Photoexcitation of Embedded Au Nanoparticles

**DOI:** 10.1021/acsphotonics.3c00184

**Published:** 2023-05-03

**Authors:** Eleonora Spurio, Jacopo Stefano Pelli Cresi, Giuseppe Ammirati, Samuele Pelatti, Alessandra Paladini, Sergio D’Addato, Stefano Turchini, Patrick O’Keeffe, Daniele Catone, Paola Luches

**Affiliations:** † Dipartimento FIM, Università degli Studi di Modena e Reggio Emilia, Via G. Campi 213/a, 41125 Modena, Italy; ‡ Istituto Nanoscienze, CNR (NANO-CNR), Via G. Campi 213/a, 41125 Modena, Italy; § Elettra-Sincrotrone Trieste, 34012 Basovizza, Trieste, Italy; ∥ CHOSE (Centre for Hybrid and Organic Solar Energy), Department of Electronic Engineering, University of Rome Tor Vergata, Via del Politecnico 1, 00133 Rome, Italy; ⊥ Istituto di Struttura della Materia − CNR (ISM-CNR), EuroFEL Support Laboratory (EFSL), 00133 Rome, Italy; # Istituto di Struttura della Materia − CNR (ISM-CNR), EuroFEL Support Laboratory (EFSL), Monterotondo Scalo 00015, Italy

**Keywords:** cerium oxide, metal nanoparticles, femtosecond transient absorption spectroscopy, localized surface plasmon resonances

## Abstract

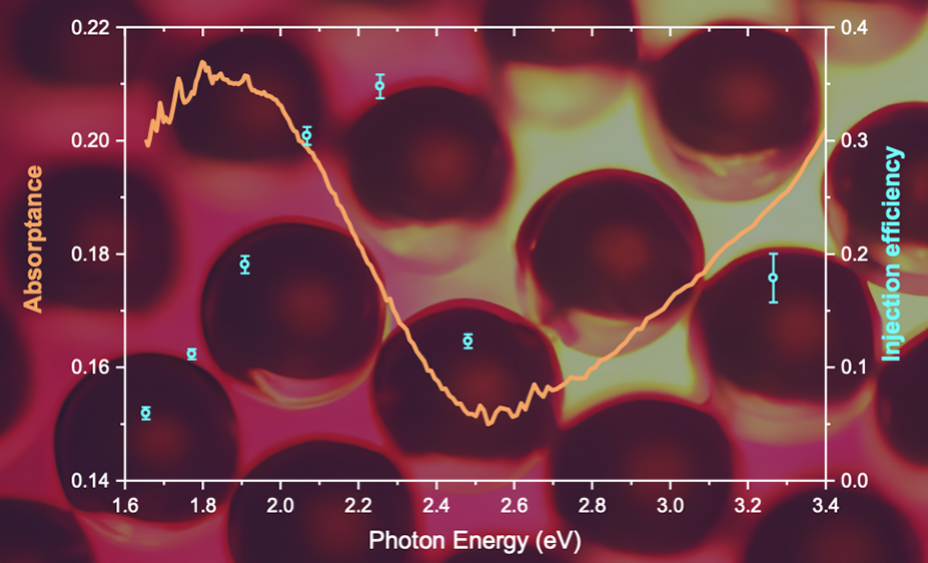

The electron injection efficiency and the steady state absorptance at different photon energies for a composite system made of Au NPs embedded in a cerium oxide matrix are reported. Cerium oxide can be coupled with plasmonic nanoparticles (NPs) to improve its catalytic properties by visible-light absorption. The present work is a study of the ultrafast dynamics of excited states induced by ultraviolet and visible-light excitation in Au NPs combined with cerium oxide, aimed at understanding the excitation pathways. The data, obtained by femtosecond transient absorption spectroscopy, show that the excitation of localized surface plasmon resonances (LSPRs) in the Au NPs leads to an ultrafast injection of electrons into the empty 4f states of the surrounding cerium oxide. Within the first few picoseconds, the injected electrons couple with the lattice distortion forming a polaronic excited state, with similar properties to that formed after direct band gap excitation of the oxide. At sub-picosecond delay times, we observed relevant differences in the energetics and the time dynamics as compared to the case of band gap excitation of the oxide. Using different pump energies across the LSPR-related absorption band, the efficiency of the electron injection from the NPs into the oxide was found to be rather high, with a maximum above 30%. The injection efficiency has a different trend in energy as compared to the LSPR-related static optical absorptance, showing a significant decrease in low energies. This behavior is explained considering different deexcitation pathways with variable weight across the LSPR band. The results are important for the design of materials with high overall solar catalytic efficiency.

## Introduction

In recent years, concern for global warming has driven intense research activities toward the study of materials suitable to act as active catalysts for environmental protection, green fuel synthesis, and sustainable energy conversion. Oxides represent abundant and stable materials largely applied to convert solar to chemical energy via various photocatalytic reactions. The optimization of the materials and of their functionality requires a detailed description of the elementary processes that underlie the energy conversion processes, namely, light absorption, charge carrier generation and transport, interaction of reactants with the catalyst surface, and generation of products.^1^

Thanks to its high reducibility, cerium oxide (CeO_2_) is a very important catalytic material,^2^ but its efficiency as a solar photocatalysts is limited by its band gap in the ultraviolet range.^3^ Different strategies can be applied to sensitize CeO_2_ to visible radiation, such as doping,^4^ nanostructuring,^5^ and coupling with plasmonic nanoparticles (NPs), like Au, Ag, or Cu.^6−8^ The latter composite systems take advantage of the large absorption and scattering cross section in the visible range, typical of plasmonic NPs. The strong interaction with the incoming radiation triggers localized surface plasmon resonances (LSPRs) in the NPs, i.e., collective resonant excitations of charges, which can relax through energy or charge transfer from the NPs to the surrounding oxide.^9,10^ Different competing mechanisms are involved in the deexcitation of LSPRs and in the activation of the nearby semiconducting oxide. In general, LSPRs decay via the generation of charges with a broad energy distribution, some of which can be injected over the Schottky barrier toward the surrounding oxide. The process competes with a second mechanism in which the LSPR energy directly excites charge carriers into empty conduction band states in the oxide across the interface, leaving positive charges in the metal valence band.^9,11,12^ These two mechanisms are expected to involve different timescales as the direct injection occurs within the first tens of femtoseconds after LSPR excitation, i.e., on the timescale of dephasing of the plasmon, while the hot electron indirect injection requires a few hundreds of femtoseconds as it must occur before the hot electrons cool by electron–electron scattering.^10^ On the picosecond timescale, electron–phonon scattering, leading to local thermal activation of the catalyst, prevails.^10^ The processes and their efficiency strongly depend on different variables, including the energy alignment of filled and empty bands of the two materials, the height and width of the Schottky barrier between them, and the NP size, shape and density.

An experimental assessment of the processes that follow LSPR excitation in materials, which combine plasmonic NPs and oxides, is presently quite challenging because the possible deexcitation pathways are quite complex and often superposed in space and time. However, efforts in this direction can drive the optimization of photocatalysts based on such materials. Several studies have revealed an enhanced activity in photocatalysts incorporating plasmonic NPs,^8,10,13−16^ but only a limited number have tried to isolate the different activation mechanisms based on the dynamics of excited states.^9,13,17^ In a previous study on CeO_2_ coupled with Ag NPs using femtosecond transient absorption spectroscopy (FTAS), we have identified an efficient and persistent plasmon-mediated electron injection from the Ag NPs to cerium oxide.^6^ However, in the case of Ag NPs, the investigation of the electron injection dynamics at ultrashort timescales was hindered by the superposition between a photoinduced absorption (PIA) signal, characteristic of CeO_2_ excitation, and the plasmon-related transient absorption (TA) signal. An element-specific analysis of the process using free-electron laser based pump-probe X-ray absorption spectroscopy allowed us to unambiguously identify a reduction of CeO_2_ compatible with a plasmon-mediated transfer of electrons into Ce 4f levels and to estimate an upper limit of 200 fs for the injection time,^18^ a value short enough to exclude thermal effects. Time-resolved photoemission spectroscopy was used to investigate the lifetimes of the photogenerated holes in the same system, finding a value of 100 ps for band gap excitation and of 300 ps in the case of LSPR excitation.^19^ The LSPR-related TA band in Ag NPs is partially overlapped with the TA signal related to electron injection, so an analysis of the injection dynamics was not possible.^6^ Since the LSPR-related TA band in Au NPs is centered at a lower energy as compared to Ag NPs, the TA signal related to electron injection could be clearly separated from the LSPR-related signal, allowing us to investigate the injection dynamics. Cu NPs show an LSPR band at a comparable energy to Au NPs; however, the interface between Cu NPs and cerium oxide is expected to be significantly less ideal than in the case of non-reactive Au NPs. For these reasons, the present work is focused on Au NPs combined with CeO_2_, a system that allowed us to obtain previously unavailable information on the dynamics of injected electrons at ultrashort times after LSPR excitation. Moreover, the injection efficiency across the LSPR absorption band has been found to have a different trend with excitation energy as compared to the static optical absorptance.

## Results and Discussion

The samples for the present study were grown by molecular beam epitaxy as described in refs (20) and (21) (see the Methods section).

**Figure 1:**
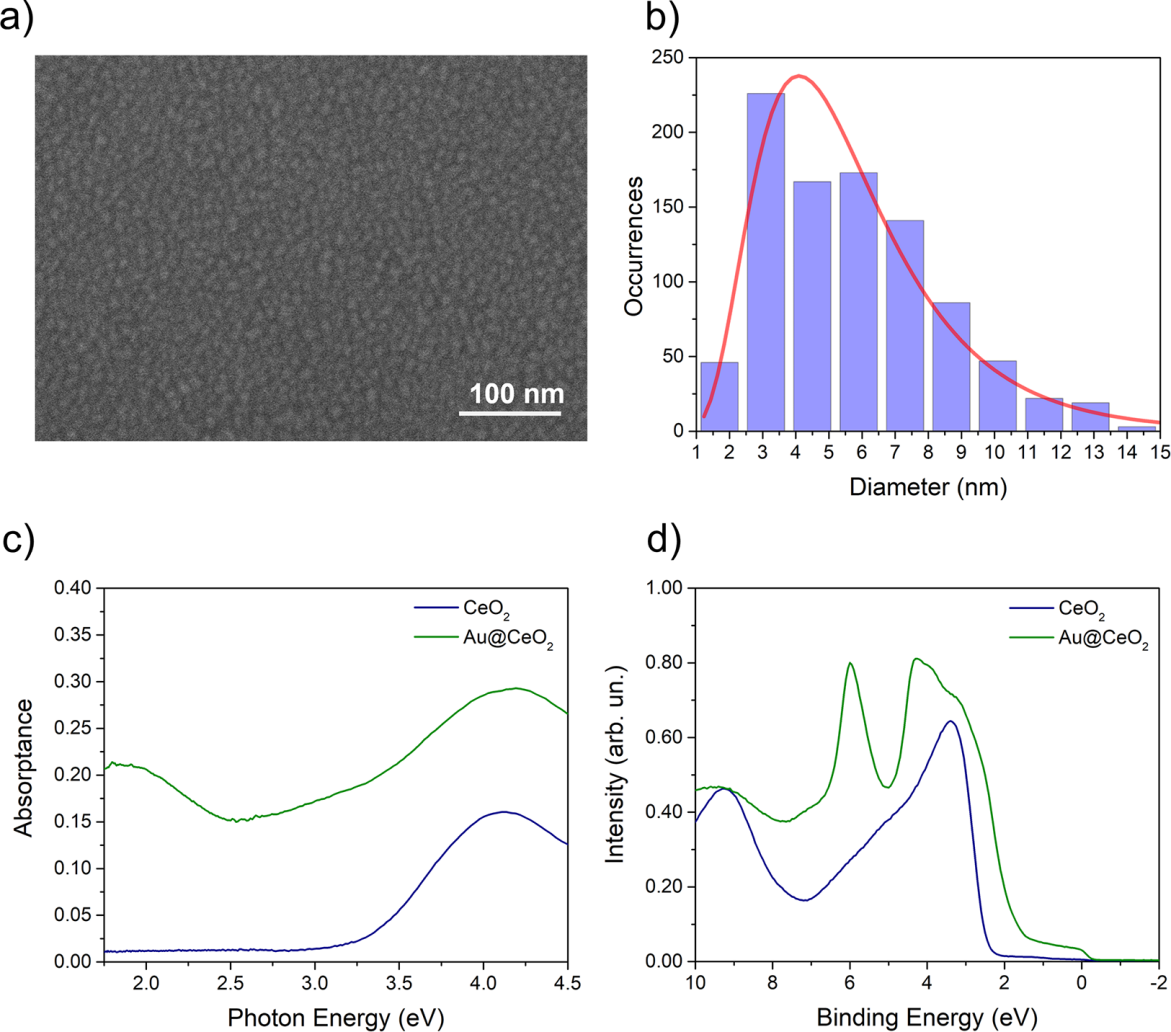
(a) SEM image of Au NPs on a CeO_2_ film and (b) size distribution extracted from the SEM images. (c) Static optical absorptance of a CeO_2_ film (blue line) and of the Au@CeO_2_ sample (green line). (d) UPS spectrum of a CeO_2_ film before (blue line) and after (green line) the growth of Au NPs.

Scanning electron microscopy (SEM) was used to characterize the morphology of the Au NPs. Figure 1a reports a representative SEM image of Au NPs on a 2 nm CeO_2_ film, showing that the Au NPs have a rather irregular shape. The Au NP size distribution is reported in Figure 1b, which shows a maximum at ∼5 nm and a full width at half-maximum (FWHM) of ∼4 nm.

**Figure 2:**
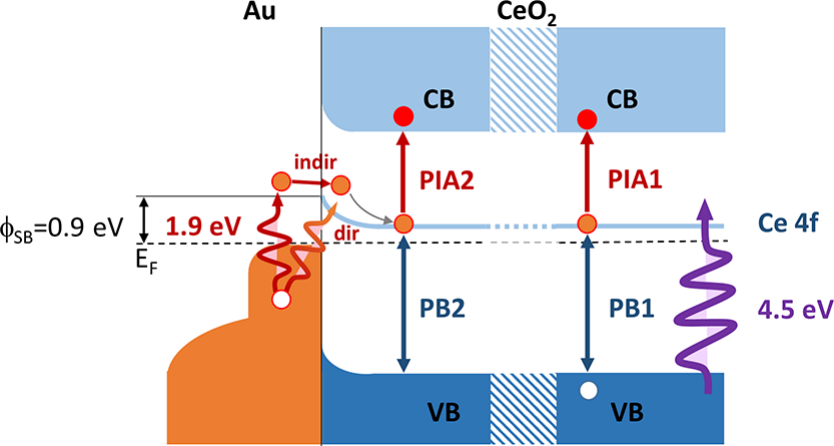
Sketch of the bands at the Au@CeO_2_ interface. The processes induced by band gap excitation (pump at 4.5 eV) and LSPR excitation (pump at 1.9 eV) and the corresponding PIA and PB signals are schematically shown.

Figure 1c shows the UV–vis optical absorptance of Au NPs embedded within two CeO_2_ films, each 2 nm thick, hereafter referred to as Au@CeO_2_, compared with a reference CeO_2_ film of 4 nm thickness. The absorptance of pure CeO_2_ exhibits a strong increase in the ultraviolet region, peaked around 4.1 eV, and a very small intensity in the visible range, in agreement with previous measurements of CeO_2_ films grown in the same conditions^6,22^ (see also the sketch in Figure 2). The incorporation of Au NPs into the oxide significantly modifies the optical absorptance of the material, with a broad band, peaked at ∼1.9 eV, appearing in the visible region due to the excitation of LSPR in Au NPs.^23,24^ The width of the observed LSPR-related band is consistent with the irregular shapes of the self-assembled NPs as measured by SEM (see Figure 1a,b and the Supporting Information, Figure S3). The absorptance also shows an increase between 2.5 and 3.2 eV, due to the excitation of interband transitions in the Au NPs.^25,26^ The excitation of sub-band gap defect states in the oxide, possibly present in the topmost CeO_2_ film above the Au NP, can also contribute to absorptance in this region. Figure 1d shows the ultraviolet photoemission spectroscopy (UPS) spectra of a 2 nm CeO_2_ film before and after Au NP deposition. The spectrum of the CeO_2_ thin film (blue line) presents a dominant feature between 2 and 7 eV due to the valence band with O 2p character. The deposition of Au NPs modifies the UPS spectrum, introducing a peak at ∼6 eV binding energy and a double peak between 2 and 5 eV binding energy, related to the Au 5d band, and a non-negligible intensity up to the Fermi edge, due to the 6s band, in analogy with bulk Au^27^ (see also the sketch in Figure 2).

**Figure 3:**
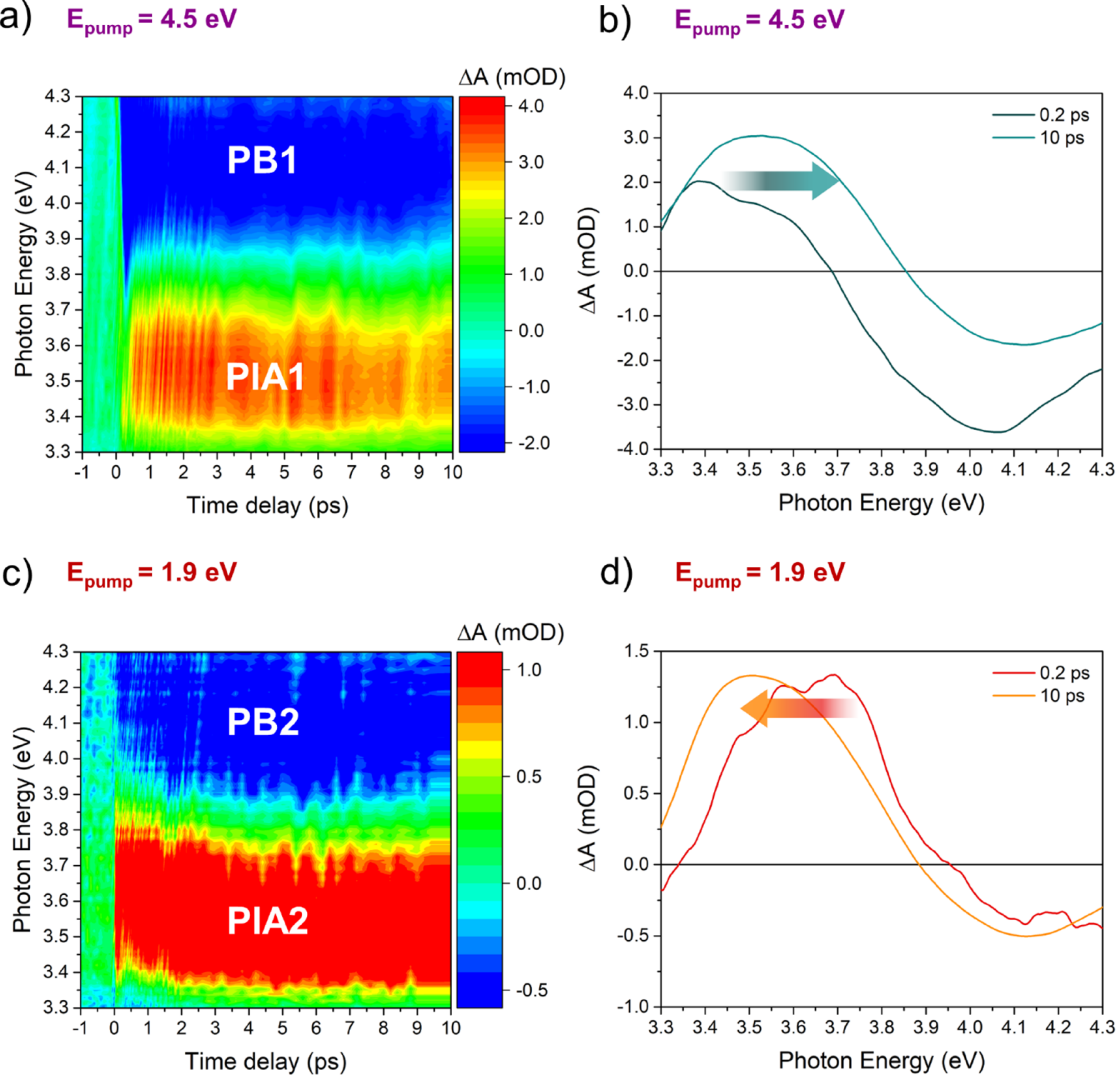
False-color map of the TA spectra of the Au@CeO_2_ sample excited with pumps at (a) 4.5 eV (above the CeO_2_ band gap) and (c) 1.9 eV (below the band gap). The PIA signals at ∼3.5 eV and the PB signals at ∼4.1 eV are indicated. (b, d) TA spectra at selected delay times of 0.2 and 10 ps for the two pump energies.

Figure 3 shows the false-color maps of the TA spectra of the Au@CeO_2_ sample, excited with a pump at 4.5 eV, above the CeO_2_ band gap (Figure 3a) and below the band gap at the LSPR of the embedded Au NPs (1.9 eV; Figure 3c), with a probe in the UV range and a delay time range limited to 0–10 ps. The maps in the full delay time range used (−1 to 300 ps) acquired with the UV and vis probe are reported in the Supporting Information, Figure S4. Figure 2b,d reports TA spectra at selected time delays. The energy and the intensity of the transient features observed and their temporal evolution provide information on the dynamics of the photoexcited states in the investigated material. For both pump energies, it is possible to clearly identify two main features in the map, one with a positive and one with a negative intensity. The positive features, labeled in Figure 2 as PIA1 and PIA2 for pump energies of 4.5 and 1.9 eV, respectively, appear at a photon energy of ∼3.5 eV, while the negative features, namely, the photoinduced bleaching (PB) and labeled as PB1 and PB2, appear at a higher energy of ∼4.1 eV. To explain the origin of PIA1 and PB1, we refer to Figure 2, which reports a sketch of the filled and empty levels at the interface between CeO_2_ and Au NPs, the energies of which are based on literature density functional theory (DFT) results,^28^ as well as on the UPS spectra shown in Figure 1d. CeO_2_ is an n-type semiconductor with a valence band (VB) with a predominantly O 2p character and a conduction band (CB) with a predominantly Ce 5d character. The energy difference between the top of the VB and the bottom of the CB is approximately 6 eV. The material is characterized by the presence of localized empty Ce 4f states between the VB and the CB that result in an optical band gap at about 4 eV.^22^ Based on previous studies on pure CeO_2_^22^ and on the same system coupled with Ag NPs,^6^ the PIA1 feature at 3.5 eV is ascribed to photoinduced absorption of VB electrons excited by the pump into empty Ce 4f levels and subsequently reexcited by the probe into the CB of Ce 5d character (Figure 2). In analogy, the PB1 feature at 4.1 eV, corresponding to the maximum of the CeO_2_ optical absorptance, as shown in Figure 1b, is ascribed to photoinduced bleaching of the VB due to an increase in the density of occupied 4f final states and to a decrease in the initial density of occupied states in the VB, as compared to the unperturbed state. The PB2 and PIA2 features in Figure 3c, acquired with an excitation energy below the band gap of CeO_2_ and resonant with the Au NPs LSPR (1.9 eV), appear at approximately the same energy as PB1 and PIA1, respectively. To understand the origin of such features, the electronic band structure of Au at the interface with CeO_2_ has to be considered (see the sketch in Figure 2). The density of states of the Au NPs investigated here, as shown by the UPS measurements reported in Figure 1d, is not very different from that of bulk Au, having a relatively low intensity 6s band extending within the first 2 eV below the Fermi level and a more intense 5d band with an onset at a binding energy of ∼2 eV. The alignment between metal and oxide energy levels at the interface critically depends on the interface properties and on possible charge transfers between the two materials. In the literature, Au NPs grown on CeO_2_ have shown a negligible steady state charge transfer,^29^ a result that is confirmed also by DFT calculations,^30^ in contrast to the case of Ag^21^ or Pt NPs^32^ in which electrons are transferred from the metal to cerium oxide when they come into contact. The empty Ce 4f levels are very close to the Fermi level, and thus, an upward band bending of the oxide CB and VB is expected at the interface with Au.^7^ The Schottky barrier, ϕ_SB_ in Figure 2, between the metal and the oxide is approximately 0.9 eV.^31^ The LSPR, excited in the Au NPs by the pump at 1.9 eV, can decay via the generation of hot electrons with an energy distribution, which extends up to the value used for LSPR excitation (1.9 eV). The most energetic electrons have enough energy to be transferred over the approximately 0.9 eV high Schottky barrier by indirect transfer (path indicated as indir in Figure 2). Alternatively, the LSPR can decay by a direct plasmonic coupling across the chemical interface^11^ in which the net effect is a transfer of electrons from the Au VB to the Ce 4f levels (path indicated as dir in Figure 2). Both the PIA2 and the PB2 signals can be assigned to the transient occupation of Ce 4f levels caused by LSPR-mediated electron injection from the NPs to the semiconductor. While the PIA2 results from the excitation of the electrons injected into the 4f states to the CB, the PB2 signal is generated by the lower density of empty final states available for the VB to 4f transition, as compared to the unperturbed state (Figure 2). We note that the intensity of the TA features observed after LSPR excitation is expectedly lower than in the case of band gap excitation due to the lower efficiency of the LSPR-mediated charge transfer process as compared to direct band gap excitation.

**Figure 4:**
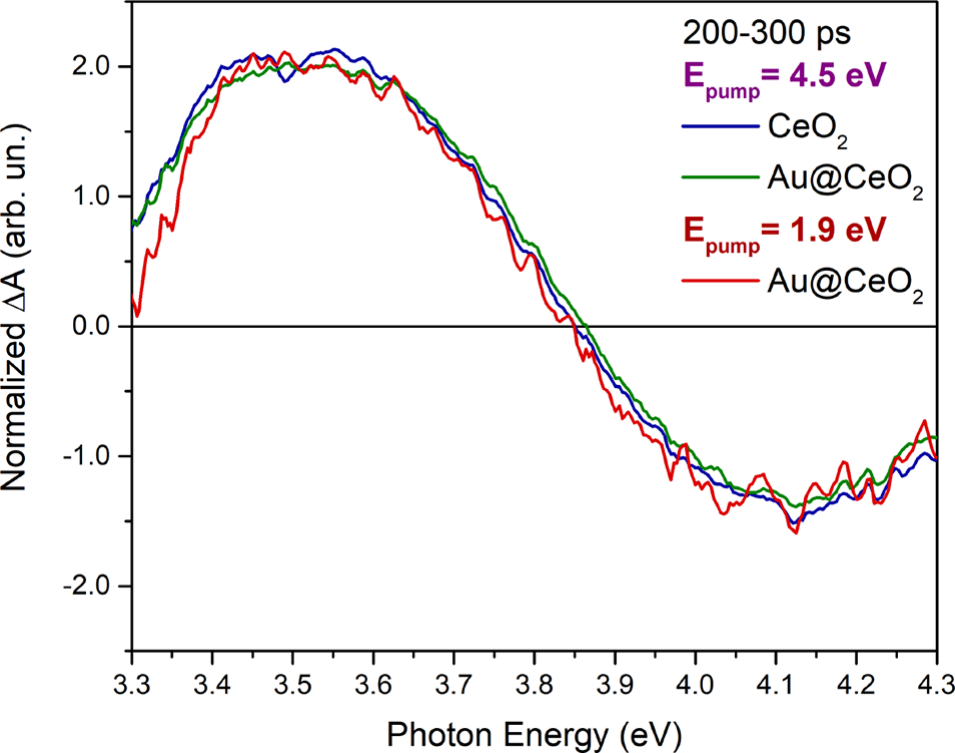
Normalized TA spectra averaged over 200–300 ps of Au@CeO_2_ (green line) and CeO_2_ (blue line) samples excited with a pump at 4.5 eV (above the CeO_2_ band gap) and of the Au@CeO_2_ sample excited with a pump at 1.9 eV (red line).

Before discussing the details of the time dependence of the transient signals, we focus on their spectral shapes at long time delays, when the systems have relaxed for hundreds of picoseconds after photoexcitation. In Figure 4, the comparison of the normalized transient signals averaged over 200–300 ps time delays is shown, following excitation of (i) CeO_2_ at 4.5 eV (blue line), (ii) Au@CeO_2_ at 4.5 eV (green line), and (iii) Au@CeO_2_ at 1.9 eV (red line), revealing the same spectral responses in the three cases. This shows that the intermediate excited states reached by the Au@CeO_2_ system within a few hundreds of picoseconds are the same for the two pump energies used. Moreover, it has the same spectral shape as that obtained on a pure CeO_2_ sample excited above the band gap. Based on the analysis presented in ref (22), which shows that the above band gap photoexcitation in a pure cerium oxide results in the formation of a small polaron in the excited state, we conclude that both the above gap and LSPR excitation in the Au@CeO_2_ system result in the formation of a small polaron in analogy with pure CeO_2_. This hypothesis is also consistent with the kinetic behavior of the PIA1, PIA2, PB1, and PB2 signals, which show a non-negligible intensity at all delay times investigated, in close analogy with CeO_2_^24^ and Ag@CeO_2_,^6^ because of the relatively long recombination times of photoexcited carriers in the oxide. Therefore, Au NPs do not introduce different recombination channels within the investigated delay time range.

**Figure 5:**
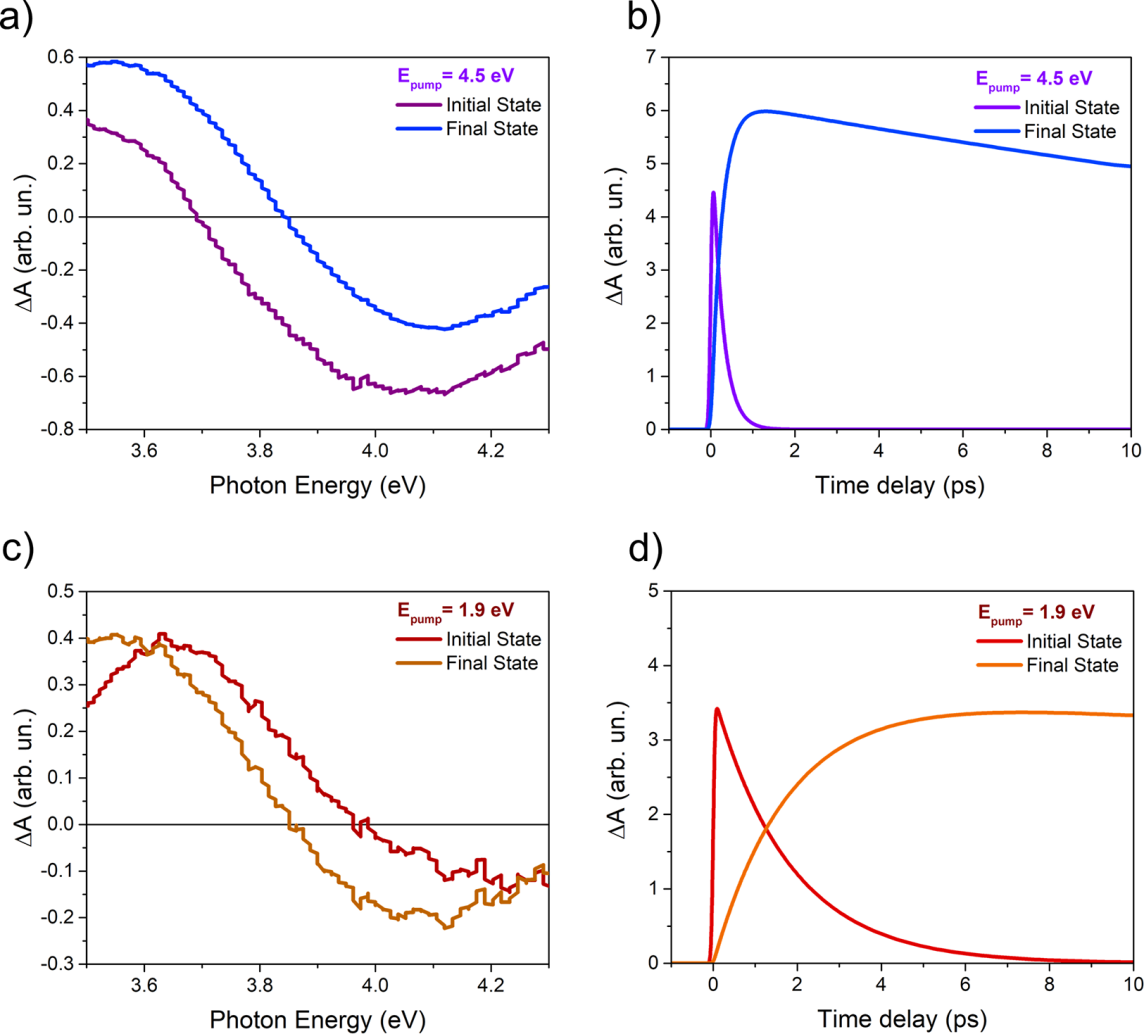
(a) Spectral components obtained from the global analysis for the pump at 4.5 eV; (b) weight dynamics of the two spectral components in panel (a); (c, d) same as panels (a, b) for the pump at 1.9 eV.

To analyze the kinetics of the TA signals in detail, we used a global analysis approach (CarpetView software package) that allowed us to extract the transient photoexcited components from the TA spectra with their temporal evolution. As shown in Figure 3b,d, the TA spectra show non-negligible modifications in energy and intensity within the first few picoseconds after excitation for both pump energies. Following the approach used in ref (22), the temporal evolution of the spectra was modeled using two components, an initially photoexcited state and a long living final state. The shape and the exponential decay/rise constants of the two components are left as free fitting parameters. The components obtained using this approach and the sequential exponential dynamics of their intensity for the two pump energies are shown in Figure 5. In analogy with the case of pure ceria^22^ for the pump at 4.5 eV, the two components are interpreted as being due to the initially populated excited 4f states that relax into a polaronic state at lower energy, resulting in a higher energy PIA (see the arrow in Figure 3b and Figure 5a). In this model, the PB remains at the same energy, although it may appear to shift due to the overlap with the PIA. As shown in Figure 5b, for the pump above the band gap, the initial state decays into the final state within the first picosecond. Overall, the dynamics of the excited states of the Au@CeO_2_ system pumped above the band gap shows the same trend as that of bare ceria,^22^ consistent with the expected negligible modifications induced by the presence of Au NPs on the electronic structure of the ceria matrix. The situation is significantly different in the case of plasmonic excitation of the Au@CeO_2_ system shown in Figure 5c, in which the PIA is initially at a higher energy than in the final state, contrary to what was observed in the case of excitation above the ceria gap (see the arrow in Figure 3d). As shown in Figure 5d, in this case, the time required to evolve from the initial to the final state is longer (more than 4 ps) than for the above band gap excitation (∼1 ps). Both of the LSPR-mediated injection processes (dir and indir) occur on a much faster timescale; for example, the Ag@CeO_2_ system showed a time constant shorter than 200 fs.^18^ Furthermore, the polaron state is also known to form with a time constant of approximately 300 fs after photoexcitation above the band gap.^22^ Therefore, the energetic and temporal dynamics observed here cannot be assigned to any of these processes. A possible explanation may lie in the fact that the electrons are injected into interface states, the energy of which may lead to a different energy position of the PIA maximum with respect to bulk states. The hypothesis that the intermediate occupied 4f states decrease in energy more slowly than the final 5d CB states as the excited state propagates from the interface (see also the sketch in Figure 2) is consistent with the observed overall reduction of the transition energy of the PIA at ultrashort decay times and with the longer time required to evolve into the bulk-like polaronic state, which dominates the TA spectra at long delay times.

Having demonstrated that after LSPR excitation in Au NPs, an injection of electrons to CeO_2_ actually occurs and that it leads to an excited state comparable to that populated by the above band gap excitation within a few hundreds of picoseconds, we now estimate the efficiency of the process across the LSPR absorption band. Since the PIA signal intensity at sub-band gap energies is proportional to the density of electrons in the Ce 4f levels after the excitation, the injection efficiency can be quantified by comparing the intensity of the PIA signal of the Au@CeO_2_ sample pumped at different pump energies with the intensity of the same signal of the sample pumped at 4.5 eV. Following the procedure reported in refs (6) and (24) under the assumption that each absorbed photon with energy higher than the CeO_2_ band gap excites an electron from the valence band to Ce 4f levels, the absorbed photon density is
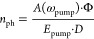
where *A*(ω_pump_) is the absorptance of the sample at the pump frequency evaluated from Figure 1c, Φ is the pump fluence, *E*_pump_ is the pump energy, and *D* is the total sample thickness.

The absorbed photon density can be correlated to the density of electrons excited in the Ce 4f levels, estimated by the integral of the intensity of the PIA signal between 50 and 250 ps. The short delay time range was not considered because the shape of the signals is affected by different shifts in energy at the different pump energies. The proportionality constant κ between the integral intensity of the PIA signal and *n*_ph_ was obtained as the ratio between these two quantities. The ratio between the κ values of Au@CeO_2_ pumped at the different energies with the reference κ measured on Au@CeO_2_ pumped at 4.5 eV corresponds to the charge injection efficiency from the Au NPs to CeO_2_ (see the Supporting Information for details).

**Figure 6:**
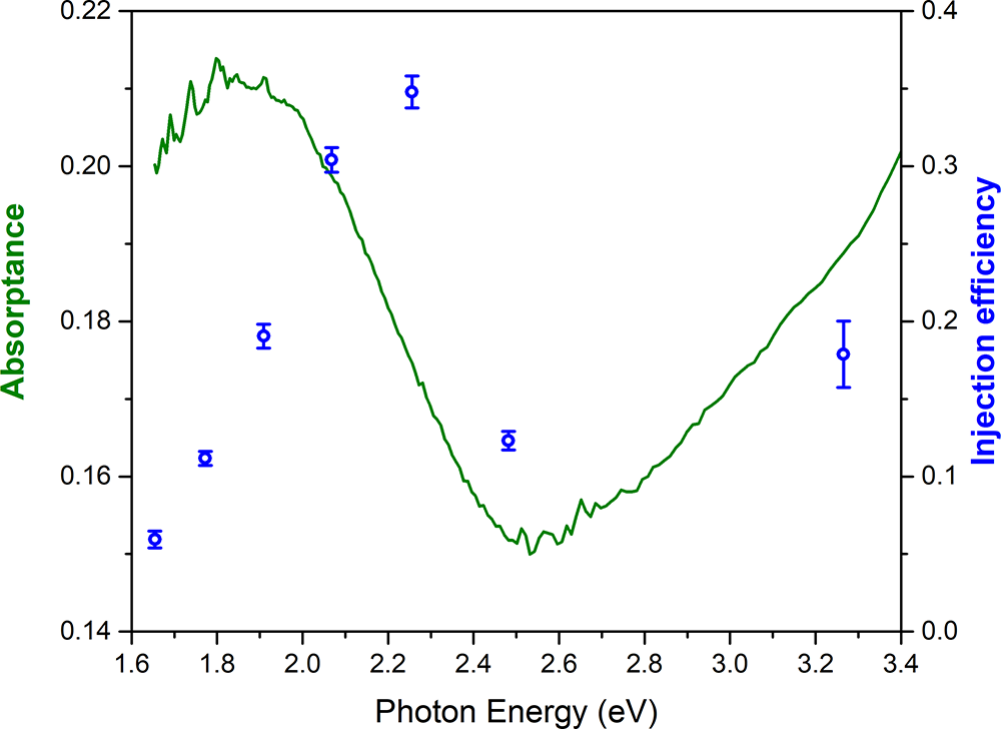
Electron injection efficiency (blue circles) as a function of the pump energy. The steady state optical absorptance (green curve) is also reported for comparison.

Figure 6 presents the electron injection efficiencies, estimated following the method described above, together with the optical absorptance of Au@CeO_2_ in the same energy region. At a pump energy of 3.3 eV, an electron injection efficiency of 18 ± 2% is assigned to the interband excitation in the Au NPs followed by an indirect injection of the resulting hot electrons over the Schottky barrier between Au and CeO_2_ (approximately 0.9 eV^31^) into the CeO_2_ CB (Figures 1d and 3). The electron injection efficiency drastically increases above 35 ± 1% at 2.25 eV, an energy that excites the high energy wing of the LSPR of the Au NPs, and it gradually decreases at lower pump energies, reaching a value below 10% at 1.6 eV, showing a trend that does not follow the intensity of the LSPR absorptance. Indeed, the indirect injection is expected to decrease its efficiency as the photon energy is decreased. The observed non-monotonic trend of the injection efficiency with energy may be due to the additional action of the LSPR-mediated direct mechanism (see also Figure 2) that enhances the electron injection in the 1.90–2.25 eV energy range, while at lower pump energies, the reduced efficiency is due to the lower probability to inject excited electrons into the semiconductor.

We note that a mismatch between the injection efficiency and the intensity of the LSPR resonance was observed also in the case of Au NPs coupled with TiO_2_^24,32^ and for Ag NPs coupled with cerium oxide.^6^ As CeO_2_ is a reducible oxide, the efficient charge transfer induced by visible-light absorption is expected to have a relevant effect on the oxide catalytic properties. The presence of extra charge in the 4f states is in fact predicted to decrease the oxygen vacancy formation energy and in turn to increase the redox activity. The low recombination rate of the excited charge within times of the order of hundreds of picoseconds is in agreement with the hole lifetimes observed on the similar system made of Ag NPs^33^ on CeO_2_.^19^ We emphasize that this aspect is very promising in the view of obtaining an efficient photocatalytic material, in which the plasmon-induced thermal activation, expected to take place in a nanosecond time regime, can be enhanced by favorable persistent electronic modifications. Further conclusions of this work are that interband transitions in gold are less efficient at transferring charge to the ceria than LSPR-mediated excitations and that increasing the overlap of the LSPR with the solar spectrum at lower energies will not increase the overall solar efficiency.

It has to be noted that cerium oxide combined with plasmonic Au nanoparticles finds wide applications, not only in heterogeneous photocatalysis and photoelectrochemistry, e.g., for selective oxidation of alcohols,^7,33^ photodegradation of organic pollutants,^34^ and photocatalytic CO_2_ reduction,^35^ but also in nanomedicine, as an innovative drug with combined antioxidant and photothermal properties.^36,37^ An improved description of the processes that follow visible-light excitation is therefore beneficial in the view of an optimized design of this class of materials in various fields of application.

## Conclusions

We investigated the dynamics of photoexcited states in a composite system made of Au NPs embedded within a thin film of cerium oxide. The optical absorptance of the sample presents a broad band in the visible range that is assigned to the LSPR excitation in the NPs. FTAS measurements showed that LSPR excitations in the Au NPs relax by transferring electrons to the surrounding semiconductor. At ultrashort delay times, below a few picoseconds, a markedly different energetic and temporal dynamics is observed in the case of LSRP-mediated injection as compared to direct band gap excitation. The observed differences are compatible with the expected modifications of the electronic properties at the interface between the metal NP and the oxide. Interestingly, after a few picoseconds, both the band gap and LSPR excitation lead to the same bulk-like polaronic state. By comparing the injection efficiencies following interband and LSPR excitations, we suggest that the interband injection is dominated by the indirect mechanism while the LSPR-mediated injection takes place by both direct and indirect mechanisms. The electron injection efficiency following LSPR excitation shows a maximum over 30% at 2.25 eV, an energy that does not correspond to the maximum of LSPR-related optical absorptance, suggesting that the injection efficiency does not simply follow the intensity of the plasmonic excitation.

## Methods

The samples for the present study were grown in an ultrahigh vacuum (UHV) apparatus (*P*∼10^–10^ mbar), which consists of two connected UHV chambers: an evaporation chamber, equipped with evaporators and gas lines for reactive molecular beam epitaxy (MBE) growth, and a chamber equipped with facilities for substrate preparation, X-ray photoemission spectroscopy (XPS), and ultraviolet photoemission spectroscopy (UPS). The use of different substrates was necessary to apply the different characterization techniques. For UV–vis spectrophotometry and femtosecond transient absorption spectroscopy (FTAS) measurements, a quartz substrate, ensuring a good optical transparency in the visible range, was used. The sample consists of a layer of Au NPs of 2 nm nominal thickness, embedded between two CeO_2_ thin films of 2 nm thickness, referred to as Au@CeO_2_. A pure CeO_2_ film of 4 nm thickness was also grown on quartz for reference. For morphological characterization using SEM, a Si substrate with thermal oxide was used to assure a high electric conductivity. A CeO_2_ film of 2 nm thickness and a layer of Au NPs of 2 nm nominal thickness were grown on top of it. A metallic Pt(111) substrate was necessary for UPS measurements since even mild charging effects had to be avoided. The quartz and Si substrates were cleaned by a 5 min bath in acetone at 423 K and by two subsequent ultrasonic baths in acetone and in isopropanol at 353 K for 3 min each. The Pt(111) substrate was prepared by cycles of sputtering (1 keV and 1 μA) and annealing (1040 K) until the surface contamination was below the XPS detection limit. The CeO_2_ thin films were grown as described in ref (20) by reactive evaporation of Ce, using an e-beam evaporator, in an oxygen partial pressure of 10^–7^ mbar. Au was evaporated from a Knudsen cell and spontaneously self-assembled into NPs, as expected for late transition metals grown by physical synthesis methods on oxide surfaces.^38^ The thickness of the film and the nominal thickness of the metal, which determines the size distribution and the density of NPs,^21^ were calibrated using a quartz microbalance. The sample morphology, and in particular the size, shape, and density of Au NPs on the cerium oxide film, is not expected to be significantly different on quartz and on Si with thermal oxide since the surface composition and roughness of the two substrates are very similar. Moreover, the surface of CeO_2_ films grown at room temperature has a rather rough morphology even on flat single crystal metal surfaces.^20^

After the growth, all the samples were characterized by *in situ* XPS to obtain quantitative information on the deposited quantity of CeO_2_ and Au and on possible variations of the chemical state of cerium oxide and of the metal (see the Supporting Information, Figure S1 for details). The spectra were acquired at normal emission using Al K_α_ photons from a double-anode X-ray source and a hemispherical electron analyzer. UPS using a He lamp with He I emission and a hemispherical electron analyzer at normal emission was used to assess the valence band density of states at the different stages of growth. Information on NP morphology was obtained using SEM (FEI Nova NanoSEM 450) on the sample grown on the Si substrate. The GMS3 GATAN software by DigitalMicrograph was used to obtain the Au NP size distribution.

The optical apparatus for UV–vis spectrophotometry consists of a xenon lamp, providing white non-polarized light, an ORIEL-MS257 monochromator, a polarizer, and a silicon photodetector. The absorptance *A* of the sample is evaluated as *A* = 1 – (*T* + *R*), where *T* and *R* are the transmittance and the reflectance, respectively, i.e., the fraction of transmitted and reflected light, measured with the impinging photon beam forming an angle of 22° with the sample surface normal. The absorptance spectral shape was comparable for s- and p-polarized light (see the Supporting Information, Figure S2). In the present work, the spectra acquired with s-polarized light, having a higher signal-to-noise ratio, were reported.

FTAS measurements were performed using a femtosecond laser system described in detail elsewhere.^39,40^ As an optical pump, a laser pulse generated by an optical parametric amplifier was tuned either to an energy above the band gap of ceria or to energies in the visible range across the plasmonic resonance of the embedded Au NPs. For the probe, a small portion of the fundamental (approximately 3 μJ) was passed through a BBO crystal to generate the second harmonic (400 nm) that was focused into a rotating CaF_2_ crystal to generate a supercontinuum in the UV energy range (3.50–4.35 eV). The temporal delay of the probe pulse was varied between −1 and 300 ps by varying the length of the optical path of the beam used to generate the white light. In the TA maps presented in this work, the chirp of the probe pulse was corrected. The instrument response function (IRF) was evaluated in separate experiments to be Gaussian with a FHWM of 70 fs. The pump fluences were 0.7 mJ/cm^2^ for the 4.5 and 3.3 eV energies and 1 mJ/cm^2^ for the other pump energies, which is sufficiently low not to induce melting of the nanoparticles.^41^

## Supporting Information Available

XPS analysis, aspect ratio of the Au NPs, UV–vis spectrophotometry, simulations of the polarizability of Au NPs in CeO_2_, full TA maps, and evaluation of the injection efficiency (PDF)
