# Supplementary material for: Injecting Electrons into CeO2 via Photoexcitation of Embedded Au Nanoparticles
Source: ACS Photonics. 2023 May 3;10(5):1566–74. doi: 10.1021/acsphotonics.3c00184 (PMC10197162; doi:10.1021/acsphotonics.3c00184)
Supplement: Supplementary file 1 — ph3c00184_si_001.pdf [file ph3c00184_si_001.pdf]

Supporting Information

Injecting electrons into CeO_2 via photoexcitation of embedded Au nanoparticles

Eleonora Spurio[1,2], Jacopo Stefano Pelli Cresi[3], Giuseppe Ammirati[4,5], Samuele Pelatti[1,2], Alessandra Paladini[6], Sergio D'Addato[1,2], Stefano Turchini[5], Patrick O'Keeffe[6], Daniele Catone[5], Paola Luches[2*]

[1] Dipartimento FIM, Università degli Studi di Modena e Reggio Emilia, Via G. Campi 213/a, 41125 Modena, Italy

[2] Istituto Nanoscienze, CNR (NANO-CNR), Via G. Campi 213/a, 41125 Modena, Italy

[3] Elettra - Sincrotrone Trieste, 34012 Basovizza, Trieste, Italy

[4] CHOSE (Centre for Hybrid and Organic Solar Energy), Department of Electronic Engineering, University of Rome Tor Vergata, Via del Politecnico 1, 00133 Rome, Italy

[5] Istituto di Struttura della Materia – CNR (ISM-CNR), EuroFEL Support Laboratory (EFSL), 00133 Rome, Italy

[6] Istituto di Struttura della Materia – CNR (ISM-CNR), EuroFEL Support Laboratory (EFSL), Monterotondo Scalo 00015, Italy

n. of pages: 8; n. of figures: 5; n. of tables: 1

XPS analysis

After the growth, all samples were characterized *in situ* by XPS, to obtain quantitative information on the deposited quantity of CeO_2 and Au and on possible variations of the chemical state of cerium oxide and of the metal. Ce 3d spectra were used to estimate the Ce^{3+} concentration, by fitting with Ce^{3+}- and Ce^{4+}-related components, following the procedure introduced by Skala et al. [4] The spectra and the corresponding fit of a 2 nm cerium oxide before and after the growth of Au nm are reported in Figure S1 a). In both cases the Ce^{3+}concentration evaluated by the fit was below the detection limit, indicating that the films have a good CeO_2 stoichiometry and that the Au NPs do not relevantly alter the Ce oxidation state. The Au 4f spectrum of the Au/CeO_2 sample, shown in Figure S1b, is compatible with bulk Au.

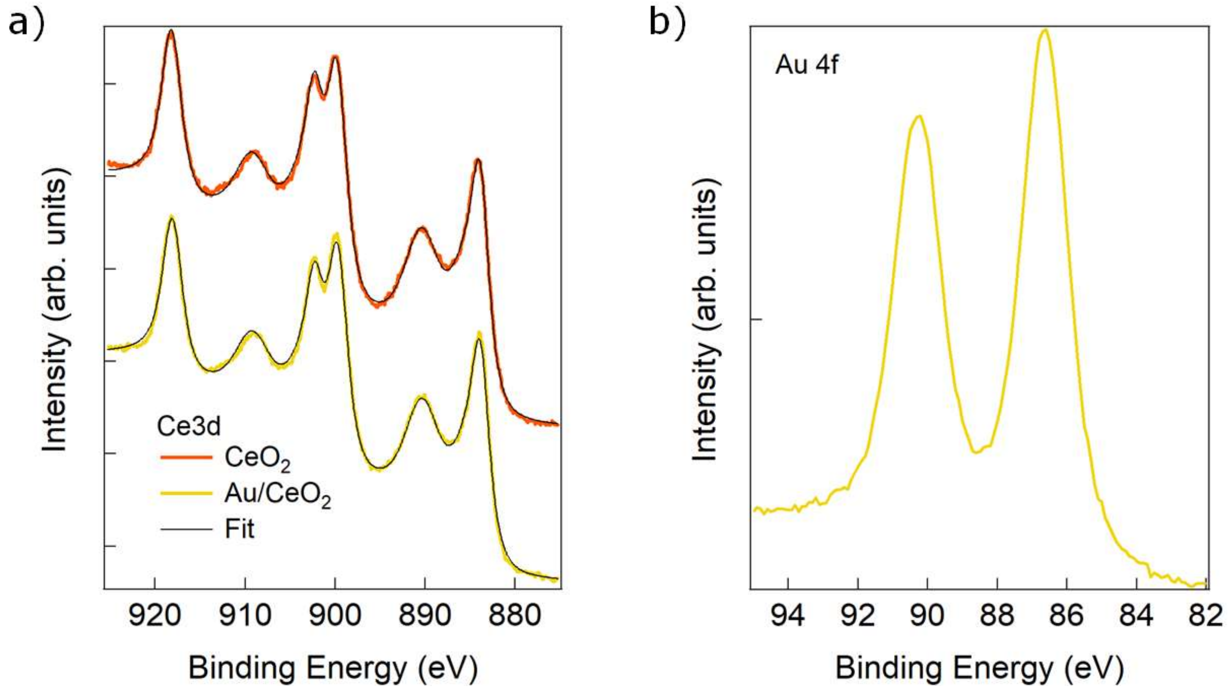

Figure S1: a) Ce 3d XPS spectra of a CeO_2 film before and after the growth of 2 nm equivalent Au NPs and corresponding fit; b) Au 4f XPS spectrum after the growth of Au NPs.

Aspect ratio of the Au NPs

The aspect ratio of the Au NPs, defined as the ratio between the in-plane average size and the out-of-plane average size, was estimated from the SEM image shown in Figure 1a of the main text. The average out-of-plane size was calculated as $\bar{h} = \frac{t_{nom}}{c}$, where t_{nom} is the nominal deposited Au thickness and the c is the fractional surface coverage. For the sample here investigated t_{nom}= 2 nm and c = 0.5, so $\bar{h}$ = 4 nm. Considering the lateral size distribution of the NPs shown in Figure 1 b), the average NP in-plane

size was calculated as 5.7 nm, which gives an average aspect ratio of 1.4. Given the width of the distribution of approximately 4 nm, and its asymmetric shape towards large in-plane NP sizes, the aspect ratio of the Au NPs in the sample ranges between approximately 1 and more than 2.

UV-Vis spectrophotometry

The static optical absorptance in the UV-Vis was measured using a Xenon lamp and a polarizer, which enables to select either p or s polarization, i.e. parallel or perpendicular to the optical plane. The polarizer enables to select either p or s polarization, i.e. parallel or perpendicular to the optical plane. The incident angle of the light was 22.5° from sample normal. In this configuration, shown in Figure S2 a), s polarization is entirely in the surface plane, while in the case of p polarization a small out-of-plane component is also present. Figure S2 b) shows the optical spectra of the Au@CeO_2 samples in the two polarizations. The shape of the spectra is the same, indicating that the samples are optically isotropic in the surface plane. The spectra acquired with s-polarized light, having a higher signal to noise ratio, are reported in the main text.

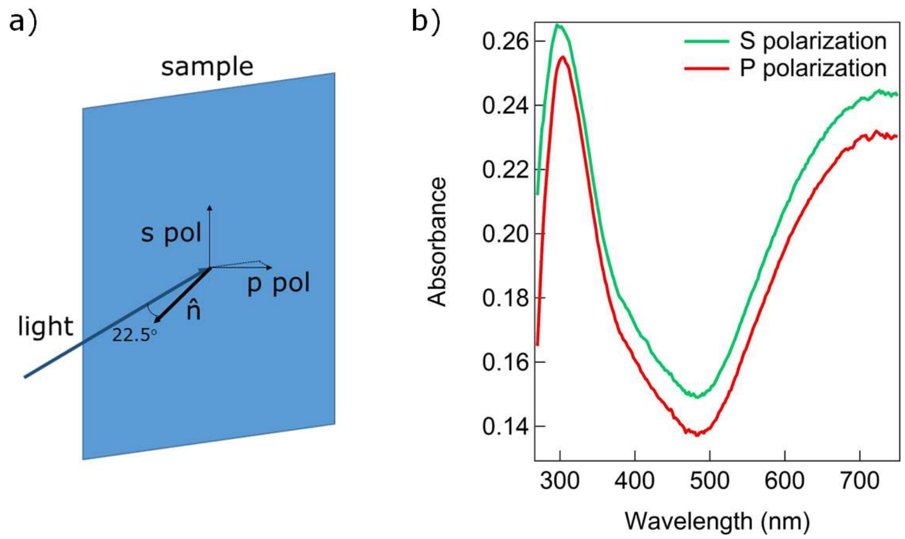

Figure S2: a) Sketch of the scattering geometry, showing the direction of the incoming light (22.5° from sample normal) and the light polarization in the case of s and p polarization; for p polarization the in-plane and out-of-plane components are also shown; b) UV-Vis absorptance spectra of the Au@CeO_2 sample in s and p polarization.

Simulations of the polarizability of Au NPs in CeO_2

The polarizability of Au NPs was simulated using the Maxwell-Garnett model, approximating the NP shape to an oblate spheroid, i.e. an ellipsoid with the three axis a, b and c following the relationship: a = b > c, where a and b are the in-plane dimensions of the nanoparticles and c is the out of plane axis.

The polarizability is given by:[5]

$$\alpha_{\perp,//}(\omega) \propto \frac{\varepsilon_{Au}(\omega) - \varepsilon_{CeO2}}{\varepsilon_{CeO2} + L_{\perp,//}[\varepsilon_{Au}(\omega) - \varepsilon_{CeO2}]}$$

where $\varepsilon_{Au}(\omega)$ is the dielectric function of Au,[6] ε_{CeO2} is the dielectric function of CeO_2,[7] $L_{\perp,//}$ are the depolarization factors in the out-of-plane and in-plane directions, given by:

$$L_x = L_y = \frac{1 - L_z}{2}$$

$$L_z = \frac{1 - e^2}{e^3}$$

e is the eccentricity of the ellipsoid:

$$e = \sqrt{\left(\frac{a}{c}\right)^2 - 1}$$

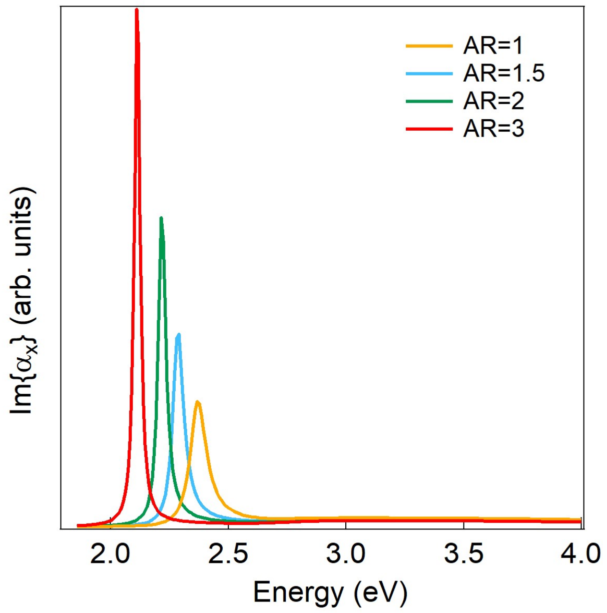

Figure S3: Imaginary part of the polarizability of Au NPs with different AR embedded in CeO_2.

and $\frac{a}{c}$ is the aspect ratio AR of the nanoparticles.

The absorption cross section of the nanoparticles is proportional to the imaginary part of the polarizability, shown in Figure S3 for different values of the NP AR. In the case of NPs with a distribution of AR values, as shown for the sample here investigated, a broad band between approximately 2 and 2.5 eV is expected.

Full TA maps

Figure S4 shows the complete false-color TA maps acquired on the Au@CeO_2 sample for the two pump energies using the UV and Vis probes in the full delay time range investigated. The LSPR-related signals, between approximately 1.6 and 3 eV decay within the first few ps for both pump energies.

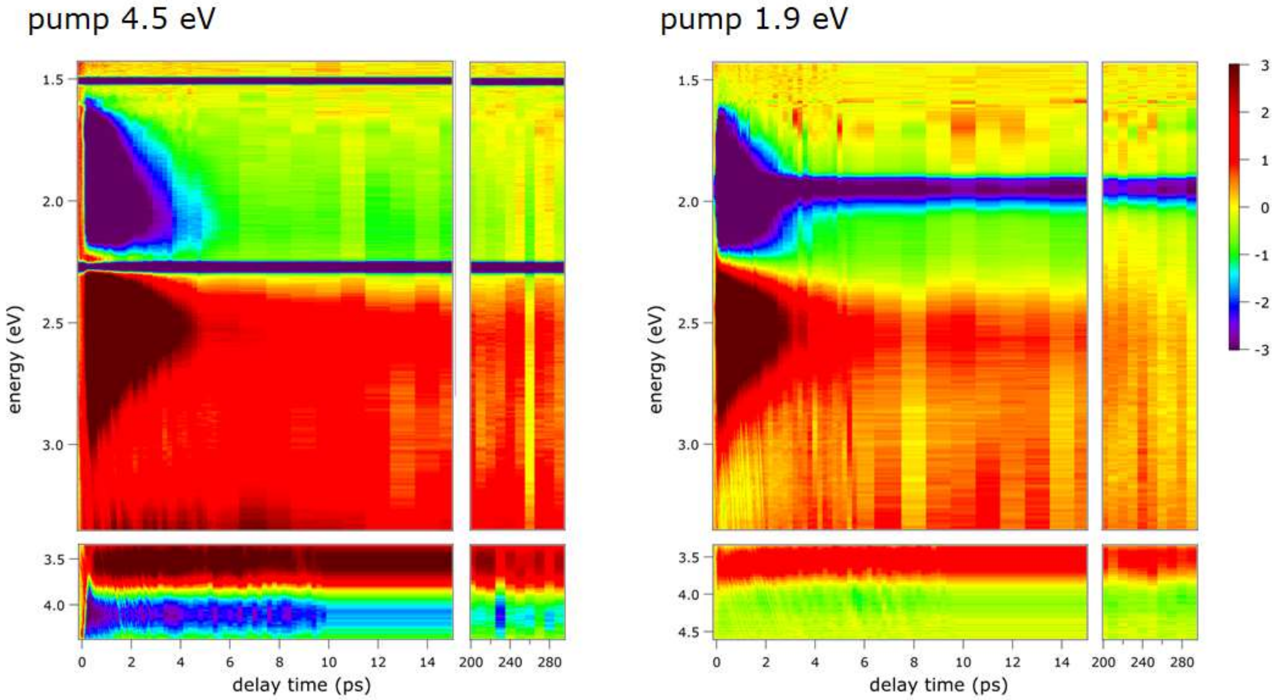

Figure S4: False-color TA maps acquired on the Au@CeO_2 sample for the two pump energies with the UV and Vis probes in the full delay time range investigated. The dark lines at 1.5 and 2.25 eV on the left map and at 1.9 eV on the right map are artifacts due to light scattered at the pump energy and at its harmonics.

Evaluation of the injection efficiency

To evaluate the injection efficiency, the ratio of the intensity of the PIA feature in the delay time range 50-250 ps and the absorbed photon density - evaluated as explained in the main text - are calculated for each pump energy, and divided by the same ratio calculated for the pump at 4.5 eV. Figure S5 shows the kinetic traces of the PIA signals at the different pump energies, obtained by averaging the TA signals within a 0.2 eV interval centered at the maximum PIA energy.

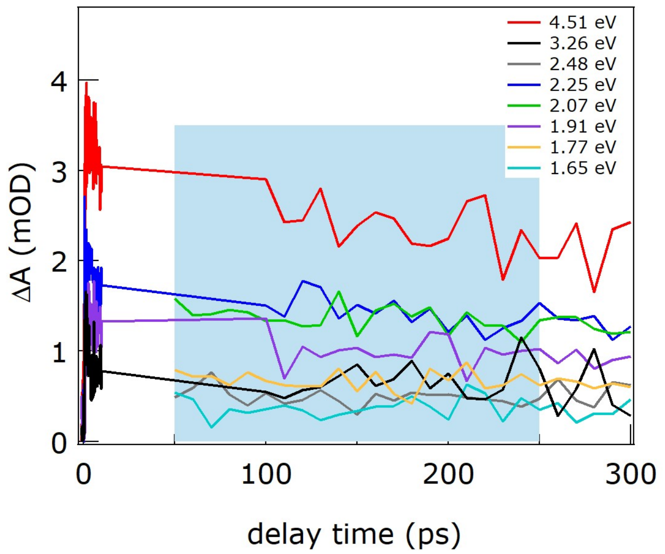

Figure S5: Kinetic traces of the PIA signal for the different pump energies. The time window used to calculate the injection efficiency is highlighted.

energy (eV)	ΔA (mOD)	n_{ph} (ph/cm^3)
4.51	2.3 ± 0.1	3.4×10^{20}
3.26	0.6 ± 0.2	5.2×10^{20}
2.48	0.49 ± 0.05	5.9×10^{20}
2.25	1.39 ± 0.02	5.9×10^{20}
2.07	1.33 ± 0.03	6.4×10^{20}
1.91	0.98 ± 0.04	7.5×10^{20}
1.77	0.65 ± 0.05	8.6×10^{20}
1.65	0.37 ± 0.05	9.2×10^{20}

Table S1: Average of the PIA signal in the 50-250 ps time range for the Au@CeO_2 sample at the different pump energies and absorbed photon density, used to evaluate the LSPR-mediated electron injection efficiency.

References

1. Luches, P.; Pagliuca, F.; Valeri, S., Morphology, Stoichiometry, and Interface Structure of CeO_2 Ultrathin Films on Pt(111). *J Phys Chem C* **2011,** *115* (21), 10718-10726.
2. Campbell, C. T., Ultrathin metal films and particles on oxide surfaces: structural, electronic and chemisorptive properties. *Surface Science Reports* **1997,** *27* (1), 1-111.
3. Luches, P.; Pagliuca, F.; Valeri, S.; Illas, F.; Preda, G.; Pacchioni, G., Nature of Ag Islands and Nanoparticles on the CeO2(111) Surface. *The Journal of Physical Chemistry C* **2012,** *116* (1), 1122-1132.
4. Skála, T.; Šutara, F.; Škoda, M.; Prince, K. C.; Matolín, V., Palladium interaction with CeO 2 , Sn–Ce–O and Ga–Ce–O layers. *Journal of Physics: Condensed Matter* **2009,** *21* (5), 055005.
5. Granqvist, C. G.; Hunderi, O., Optical properties of ultrafine gold particles. *Phys Rev B* **1977,** *16* (8), 3513-3534.
6. McPeak, K. M.; Jayanti, S. V.; Kress, S. J. P.; Meyer, S.; Iotti, S.; Rossinelli, A.; Norris, D. J., Plasmonic Films Can Easily Be Better: Rules and Recipes. *ACS Photonics* **2015,** *2* (3), 326-333.
7. Chiu, F.-C.; Lai, C.-M., Optical and electrical characterizations of cerium oxide thin films. *Journal of Physics D: Applied Physics* **2010,** *43* (7), 075104.
8. Ferrera, M.; Della Valle, G.; Sygletou, M.; Magnozzi, M.; Catone, D.; O'Keeffe, P.; Paladini, A.; Toschi, F.; Mattera, L.; Canepa, M.; Bisio, F., Thermometric Calibration of the Ultrafast Relaxation Dynamics in Plasmonic Au Nanoparticles. *ACS Photonics* **2020,** *7* (4), 959-966.
9. Catone, D.; Di Mario, L.; Martelli, F.; O'Keeffe, P.; Paladini, A.; Stefano Pelli Cresi, J.; Sivan, A. K.; Tian, L.; Toschi, F.; Turchini, S., Ultrafast optical spectroscopy of semiconducting and plasmonic nanostructures and their hybrids. *Nanotechnology* **2020,** *32* (2), 025703.
10. Magnozzi, M.; Proietti Zaccaria, R.; Catone, D.; O'Keeffe, P.; Paladini, A.; Toschi, F.; Alabastri, A.; Canepa, M.; Bisio, F., Interband Transitions Are More Efficient Than Plasmonic Excitation in the Ultrafast Melting of Electromagnetically Coupled Au Nanoparticles. *The Journal of Physical Chemistry C* **2019,** *123* (27), 16943-16950.
